# Common variant genetic background risk impacts Alzheimer's disease rare genetic variant associations

**DOI:** 10.1002/alz70855_106998

**Published:** 2025-12-24

**Authors:** Oluwatosin A Olayinka, John J. Farrell, Congcong Zhu, Zainab Khurshid, The Alzheimer's Disease Sequencing Project (ADSP), Eden R. Martin, William S Bush, Margaret Pericak‐Vance, Li‐San Wang, Gerald D. Schellenberg, Jonathan L Haines, Kathryn L. Lunetta, Xiaoling Zhang, Lindsay A. Farrer

**Affiliations:** ^1^ Boston University Bioinformatics Program, Boston, MA, USA; ^2^ Department of Medicine (Biomedical Genetics), Boston University Chobanian & Avedisian School of Medicine, Boston, MA, USA; ^3^ Biomedical Genetics, Department of Medicine, Boston University Medical School, Boston, MA, USA; ^4^ Boston University Chobanian & Avedisian School of Medicine, Boston, MA, USA; ^5^ Biomedical Genetics, Boston University Medical School, Boston, MA, USA; ^6^ Penn Neurodegeneration Genomics Center, Department of Pathology and Laboratory Medicine, Perelman School of Medicine, University of Pennsylvania, Philadelphia, PA, USA; ^7^ Perelman School of Medicine, University of Pennsylvania, Philadelphia, PA, USA; ^8^ John P. Hussman Institute for Human Genomics, Miller School of Medicine, University of Miami, Miami, FL, USA; ^9^ Dr. John T. Macdonald Foundation Department of Human Genetics, University of Miami Miller School of Medicine, Miami, FL, USA; ^10^ University of Miami, Miller School of Medicine, John P. Hussman Institute for Human Genomics, Miami, FL, USA; ^11^ University of Miami Miller School of Medicine, Miami, FL, USA; ^12^ John P. Hussman Institute for Human Genomics, University of Miami Miller School of Medicine, Miami, FL, USA; ^13^ Department of Population and Quantitative Health Sciences, School of Medicine, Case Western Reserve University, Cleveland, OH, USA; ^14^ Departments of Medicine (Biomedical Genetics), Neurology, Ophthalmology, Biostatistics, and Epidemiology, Boston University Schools of Medicine and Public Health, Boston, MA, USA; ^15^ Cleveland Institute for Computational Biology, Case Western Reserve University, Cleveland, OH, USA; ^16^ Cleveland Institute for Computational Biology, Department of Population and Quantitative Health Sciences, Case Western Reserve University, Cleveland, OH, USA; ^17^ Case Western Reserve University, Cleveland, OH, USA; ^18^ Department of Population and Quantitative Health Sciences, Case Western Reserve University School of Medicine, Cleveland, OH, USA; ^19^ Department of Population & Quantitative Health Sciences, Case Western Reserve University School of Medicine, Cleveland, OH, USA; ^20^ Department of Population and Quantitative Health Sciences, Institute for Computational Biology, Case Western Reserve University, Cleveland, OH, USA; ^21^ Cleveland Institute for Computational Biology, Department of Population & Quantitative Health Sciences, School of Medicine, Case Western Reserve University, Cleveland, OH, USA; ^22^ 1501 NW 10th Avenue, Miami, FL, USA; ^23^ Department of Neurology, University of Miami Miller School of Medicine, Miami, FL, USA; ^24^ University of Pennsylvania Perelman School of Medicine, Philadelphia, PA, USA; ^25^ University of Pennsylvania, Philadelphia, PA, USA; ^26^ Penn Neurodegeneration Genomics Center, Department of Pathology and Laboratory Medicine, University of Pennsylvania Perelman School of Medicine, Philadelphia, PA, USA; ^27^ Institute on Aging, Philadelphia, PA, USA; ^28^ Department of Pathology and Laboratory Medicine, University of Pennsylvania Perelman School of Medicine, Philadelphia, PA, USA; ^29^ Department of Pathology and Laboratory Medicine, University of Pennsylvania, Philadelphia, PA, USA; ^30^ Penn Neurodegeneration Genomics Center, Perelman School of Medicine, University of Pennsylvania, Philadelphia, PA, USA; ^31^ University of Pennsylvania Perelman School of Medicine, Path & Lab Med, Stellar Chance, Philadelphia, PA, USA; ^32^ Case Western Reserve University School of Medicine, Department of Population & Quantitative Health Sciences, Cleveland Institute for Computational Biology, Cleveland, OH, USA; ^33^ Case Western Reserve University School of Medicine, Cleveland, OH, USA; ^34^ Department of Epidemiology and Biostatistics, Case Western Reserve University, Cleveland, OH, USA; ^35^ Department of Population & Quantitative Health Sciences, School of Medicine, Case Western Reserve University, Cleveland, OH, USA; ^36^ School of Medicine, Case Western Reserve University, Cleveland, OH, USA; ^37^ Framingham Heart Study, Boston University Chobanian & Avedisian School of Medicine, Boston, MA, USA; ^38^ Framingham Heart Study, Framingham, MA, USA; ^39^ Boston University School of Public Health, Boston, MA, USA; ^40^ Department of Biostatistics, Boston University School of Public Health, Boston, MA, USA; ^41^ Boston University Department of Medicine (Biomedical Genetics), School of Medicine, Boston, MA, USA; ^42^ Boston University, Boston, MA, USA; ^43^ Bioinformatics Program, Boston University, Boston, MA, USA; ^44^ Department of Neurology, Boston University Chobanian & Avedisian School of Medicine, Boston, MA, USA; ^45^ Boston University School of Medicine, Department of Medicine, Biomedical Genetics, Boston, MA, USA; ^46^ Department of Epidemiology, Boston University School of Public Health, Boston, MA, USA; ^47^ Boston University Alzheimer's Disease Research Center, Boston University Chobanian & Avedisian School of Medicine, Boston, MA, USA; ^48^ Alzheimer's Disease Research Center, Boston University Chobanian & Avedisian School of Medicine, Boston, MA, USA, Boston, MA, USA; ^49^ Department of Neurology and Ophthalmology, Boston University Chobanian & Avedisian School of Medicine, Boston, MA, USA

## Abstract

**Background:**

Polygenic risk scores (PRS) are measures of an individual's aggregate genetic risk of disease and are typically calculated using common variants. Rare variants (RV), though not captured by PRS, have been shown to play an important role in AD. We utilized an AD PRS to discover associations with novel RVs.

**Method:**

PRS for non‐Hispanic white (NHW) samples (*n* = 11,409; AD=6630, controls=4779) from the Alzheimer's Disease Sequencing Project (ADSP) R4 dataset were calculated using published GWAS summary statistics of NHW subjects, excluding the APOE region. Participants were classified into high (*n* = 5,442) and low (*n* = 5,967) PRS groups based on the median PRS. After quality control, 11,485,531 low frequency variants (LV) (1% < MAF < 5%) and RVs with a MAC>5 were included in the analysis. The association of each variant was evaluated in low and high PRS groups separately using a regression model including covariates for age, sex, and principal components of ancestry implemented in GENESIS.

**Result:**

Genome‐wide significant (GWS) associations of RVs and LVs with AD were identified in both the low and high PRS groups. Risk variants were disproportionately enriched in the low group while protective ones were disproportionately enriched in the high group. Among the GWS, RVs in the coding region of *NYNRIN* (rs570910195) (β=4.06, *p* = 4.37x10^‐10^) and 52 kb upstream of *LOC105373398* (rs572619714, β=1.67, *p* = 2.20x10^‐8^) increased AD risk in the low PRS group. A rare deletion located 27 kb downstream of *SDHC* (chr1:161390036) was associated with decreased AD risk (β=‐2.33, *p* = 7.45x10^‐9^) and a low frequency variant 20 kb downstream of *SMNDC1* (rs150913764, MAF=0.02) was associated with increased AD risk (β=0.96, *p* = 3.13x10^‐8^) in the high PRS group. These variants were not associated with AD in the other respective PRS group.

**Conclusion:**

Stratifying individuals into those with lower and higher risk for AD based on aggregated effects of common variants enhanced the detection of RV and LV associations. Our results suggest that a PRS calculated from common variants may not necessarily correspond to an individual's genetic relative risk for AD. These findings also provide unique opportunities to study RVs whose effects are opposite to the risk conferred by the genetic background.

